# Exploratory study of the association of volumetric modulated arc therapy (VMAT) plan robustness with local failure in head and neck cancer

**DOI:** 10.1002/acm2.12099

**Published:** 2017-05-14

**Authors:** Wei Liu, Samir H. Patel, Daniel P. Harrington, Yanle Hu, Xiaoning Ding, Jiajian Shen, Michele Y. Halyard, Steven E. Schild, William W. Wong, Gary E. Ezzell, Martin Bues

**Affiliations:** ^1^ Department of Radiation Oncology Mayo Clinic Phoenix Arizona 85054 USA

**Keywords:** head and neck cancer, local failure, plan robustness, uncertainty, VMAT

## Abstract

This work is to show which is more relevant to cause local failures (LFs) due to patient setup uncertainty between the planning target volume (PTV) underdosage and the potential target underdosage subject to patient setup uncertainties in head and neck (H&N) cancer treated with volumetric‐modulated arc therapy (VMAT). Thirteen LFs in 10 H&N patients treated by VMAT were analyzed. Measures have been taken to minimize the chances of insufficient target delineation for these patients and the patients were clinically determined to have LF based on the PET/CT scan results by an experienced radiologist and then reviewed by a second experienced radiation oncologist. Two methods were used to identify the possible locations of LF due to underdosage: (a) examining the standard VMAT plan, in which the underdosed volume in the nominal dose distribution (UVN) was generated by subtracting the volumes receiving the prescription doses from PTVs, and (b) plan robustness analysis, in which in addition to the nominal dose distribution, six perturbed dose distributions were created by translating the CT iso‐center in three cardinal directions by the PTV margin. The coldest dose distribution was represented by the minimum of the seven doses in each voxel. The underdosed volume in the coldest dose distribution (UVC) was generated by subtracting the volumes receiving the prescription doses in the coldest dose distribution from the volumes receiving the prescription doses in the nominal dose distribution. UVN and UVC were subsequently examined for spatial association with the locations of LF. The association was tested using the binominal distribution and the Fisher's exact test of independence. We found that of 13 LFs, 11 were associated with UVCs (*P* = 0.011), while three were associated with UVNs (*P* = 0.99). We concluded that the possible target underdosage due to patient setup uncertainties appeared to be a more relevant factor associated with LF in VMAT for H&N cancer than the compromised PTV coverage at least for the patients included in this study.

## 1 INTRODUCTION

Volumetric‐modulated arc therapy (VMAT) is a novel form of intensity‐modulated radiotherapy (IMRT) that can deliver complex, 3‐dimensional dose distributions by using single or multiple arcs. Compared with conventional static‐field IMRT (hereafter termed *IMRT*), VMAT has the advantages of improved dose distributions, faster treatment delivery, and decreased monitor unit requirements.^1^ Therefore, VMAT has been rapidly adopted as the preferred treatment technique for head and neck (H&N) cancers.^2^

Despite improved technologies in radiotherapy for H&N cancer, local failure (LF) remains the most important cause of patient morbidity and mortality.^3^, ^4^, ^5^, ^6^ LF causes significant morbidity and often leads to death. Many factors are associated with LF after radiotherapy. In addition to biological factors inherent in the disease, technical aspects of radiotherapy may play a role in LF. To minimize severe complications such as brainstem necrosis, xerostomia, and dysphagia, radiation oncologists often reduce target margins, compromise the target coverage, or both, to avoid critical structures, resulting in a region of low dose, which in turn may cause LF.^4^

Highly conformal treatment techniques such as IMRT and VMAT are capable of generating sharp dose gradients between tumors and nearby critical structures. However, uncertainties introduced by variations in patient setup (hereafter termed patient‐setup *uncertainties*) and organ motion may lead to target underdosage, thus contributing to LF.^7^ In planning for external beam therapy, setup uncertainties and organ motion are addressed by uniform geometric expansion of the clinical target volume (CTV) to form the planning target volume (PTV).^8^ In our clinics, the PTV coverage is evaluated to assess the probability of LF due to patient setup uncertainties.^4^, ^9^ This approach assumes the static dose cloud approximation, i.e., the approximation that the dose cloud is static relative to the room‐coordinate system.^10^ During the past decade, researchers have extensively studied the sensitivity of IMRT plans to uncertainties and organ motion in H&N cancer radiotherapy.^7^, ^11^, ^12^, ^13^, ^14^, ^15^, ^16^, ^17^ However, few studies have investigated the sensitivity of VMAT plans to these uncertainties.^18^

More importantly, almost all the reported studies 7, 11, 12, 13, 14, 15, 16, 17 are computer‐based dosimetric investigations. To our knowledge, no studies have examined the association of LF observed in the clinic with VMAT plan robustness. Therefore, we evaluated data of 10 patients with H&N cancer presenting with a total of 13 LFs after being treated with VMAT in order to determine whether potential target underdosage subject to patient setup uncertainties may be a more relevant factor in predicting LF than the conventional PTV method.

## 2 METHODS AND MATERIALS

### 2.A Patient data and treatment planning

The scope of this work is limited to show which is more relevant to cause the LF due to patient setup uncertainty between the compromised PTV coverage and the potential target underdosage subject to patient setup uncertainties. Other complicated technical aspects and biological aspects including surgical seeding are not considered in this exploratory study.

In total 396 patients with H&N cancer were treated using VMAT at our institution from January 19, 2012 to August 31, 2015. All these patients had positron emission tomography/computed tomography (PET/CT) (General Electric Discovery* PET/CT 610) and/or magnetic resonance imaging (MRI) (GE Discovery MR750w) before radiation therapy to help diagnostics of the disease and facilitate the delineation of the targets. The postoperative patients were not required to have MRI. The PET/CT has the in‐plane spatial resolution of 3.6 mm with the slice thickness of 3.3 mm. The MRI has the in‐plane spatial resolution of 1.0 mm with the slice thickness of 3.0 mm. All patients had radiographic staging studies using PET/CT. Pathologic staging was used for patients treated postoperatively, whereas clinical staging was used for patients who underwent definitive treatment with radiation therapy.

10 patients with LF were identified by an experienced radiation oncologist. Characteristics of the patients, tumors, and treatment are shown in Table 1. The subsites of the tumors were oral cavity/oropharynx (seven patients), nasopharynx (two patients), and supraglottic larynx (one patient). Tumors were classified into two classes: patients with *T* > 2 or *N* ≥ 2 were considered to be locally advanced (seven patients), otherwise they were considered as early stage (three patients). None of the patients had distant metastatic disease at presentation. Nine patients had histologically verified squamous cell carcinoma (SCC) and 1 patient had adenocarcinoma. All these patients had follow‐up with PET/CT in at least 3 months after radiotherapy. Different from the planning CT simulation, the PET/CT scans are for the purpose of diagnosis and thus patients do not have any immobilization during the PET/CT. The patients were clinically determined to have LF based on the PET/CT scan results by an experienced radiologist and then reviewed by a second experienced radiation oncologist.^6^

**Table 1:** Characteristics of patients, tumors, and treatment

Patient	Location	Stage	Histology	Treatment with prescription dose (Gy/Fractions)	CTV_High_ Volume (cc)
1	Hard palate	T4bN0	ACC^a^	ChemoRT (70/35)	242
2	Nasopharynx	T1N0	SCC^a^	RT (70/33)	37
3	Base of Tongue	T2N2b	SCC	Surgery + ChemoRT (70/35)	35
4	Left buccal mucosa	T1N1	SCC	Surgery + ChemoRT (60/30)	243
5	Oral Tongue	T1N1	SCC	Surgery + RT (60/30)	47
6	Oral Tongue	T2N2b	SCC	Surgery + ChemoRT (70/35)	41
7	Base of tongue	T2/T3N2b	SCC	ChemoRT (70/35)	265
8	Nasopharynx	T4N2	SCC	ChemoRT (70/33)	79
9	Supraglottis	T2N2c	SCC	RT (66/33)	113
10	Left Mandible	T2N0	SCC	Surgery+RT (60/30)	72

a ACC, adenocarcinoma; SCC, squamous cell carcinoma; ChemoRT, chemoradiotherapy; RT, radiation therapy.

Each patient was treated using VMAT with 2 or 3 arcs. Two dose levels were prescribed and administered using a simultaneous integrated boost technique. The target region, which received a higher prescribed dose, was referred to as CTV_High_ and the region that received a lower prescribed dose was referred to as CTV_Low_. CTVs were delineated by a physician, with CTV_High_ defined as the volume with gross disease or high risk microscopic disease (gross tumor volume or postoperative tumor bed with a nonuniform 5–10 mm margin), including the high‐risk nodal region adjacent to the gross disease. This region was considered to be at risk for harboring subclinical disease. The CTVs were delineated by an experienced radiation oncologist specialized in treating H&N cancers. A contour review by a second radiation oncologist was performed to ensure target volumes were clinically acceptable based on review of the clinical notes including the operative note if applicable and radiologic imaging (PET/CT and/or MRI) to minimize the chances of insufficient target delineation. The volumes of CTV_High_ varied from 35 to 265 cc (median 75.5 cc). CTV_Low_ typically encompassed a 10–15 mm margin beyond CTV_High_ and low‐risk nodal regions. PTV_High_ and PTV_Low_ were formed by uniformly expanding the corresponding CTV by 3 mm. None of the radiation treatment volumes were cropped further because of the proximity of nearby organs at risk except that PTVs were cropped at least 3 mm from the skin if needed. The prescription doses and fractionation scheme varied (Table 1).

Doses to critical normal structures were constrained to meet acceptable tolerance dose values whenever possible as defined in the departmental H&N cancer treatment protocol (Table S1). All VMAT plans were generated by experienced dosimetrists or physicists using the treatment planning software Eclipse^TM^ (Varian Medical Systems, Palo Alto, CA, USA) and were approved by the treating physician. A second review by a radiation oncologist was performed to verify that all plans met departmental criteria (Table S1).

### 2.B Underdosed volume because of cold spots in the PTVs in the nominal dose distribution (standard method to assess cold spots)

In photon radiation therapy, the dose distributions of PTVs are usually used to evaluate the impact of patient setup uncertainties. Two dose**–**volume histogram (DVH) indices were used to assess the PTV coverage: D_95%_, the dose covering 95% of the PTV and V_95%,_ the subvolume of the PTV receiving 95% of the prescription dose.

Cold spots within the PTVs are possible sources of LF. The underdosed volume in the nominal dose distribution (UVN) due to the cold spots within the PTVs was generated by subtracting the volume that received prescription doses (V_100%_) from the PTVs: UVN = PTV – V_100%_.

### 2.C Modelling of patient setup uncertainty and resultant underdosed volume (using robustness analysis to assess the potential cold spots)

A coldest setup deviation dose distribution was computed as follows: interfractional patient setup uncertainties were modeled by applying both positive and negative shifts of the patient's CT iso‐center in the antero‐posterior (A‐P), superior‐inferior (S‐I), and lateral (L‐R) directions by the same distance used for defining the PTV margins (3 mm). The original VMAT plans were used for all recalculations and yielded seven (nominal plus six perturbed) dose distributions per plan for each patient. The coldest dose distribution was then represented by the minimum of the seven doses in each voxel. Although the coldest dose distribution might not be realistic, it served as a lower bound for the coldest possible dose coverage. From ICRU No. 50 the PTV margin is chosen with the implicit assumption that the CTV will remain covered with the prescribed isodose surface with high probability (e.g., 95%) in the presence of uncertainties. This is a good assumption for photons since, as pointed out by Meleike et al., (2006),^12^ the spatial nature of photon dose distributions is minimally perturbed by uncertainties. Actually from the definition of PTV, PTV can be considered to be the worst‐case of CTV in the presence of patient setup uncertainties and it is also not realistic since CTV cannot reside in the top and the bottom of PTV simultaneously. Ideally if the static dose cloud approximation (the assumption for the PTV concept to work to mitigate the impact of patient setup uncertainties) is rigidly satisfied, the coldest dose distribution of CTV should be equivalent to the nominal dose distribution of PTV. The value of 3 mm is chosen as it is the PTV margin size used for these patients.

The coldest dose distribution subject to uncertainty was then imported to Eclipse^TM^ and overlaid on the planning CT. The underdosed volume in the coldest dose distribution (UVC) was generated by subtracting the V_100%_ in the coldest dose distribution from the V_100%_ in the nominal dose distribution: UVC=V_100%_|_nominal_‐V_100%_|_coldest_. The way in this work to define the UVC using the coldest dose distribution is to follow the same logic of the PTV definition, thus leading to fair comparison with the UVN.

### 2.D Association of underdosed volumes with LF

All patients had PET/CT scans performed at least 3 months after radiotherapy. Each patient had LF within and around CTVs (both CTV_High_ and CTV_Low_), and three patients (patient # 4, 6 and 7, Table 2) had a second LF after salvage radiotherapy (total of 13 instances of LF). Failures were categorized as in‐field, marginal, or out‐of‐field if > 95%, 20%–95%, or < 20% of the LF was within the 95% prescription iso‐dose lines.^3^

**Table 2:** Local failure (LF) location, type of LF and V_95%_, D_95%_ normalized by the prescription doses

Patient	LF Location	Type of LF	D_95%_ (%)		V_95%_ (%)	
			PTV_High_	PTV_Low_	PTV_High_	PTV_Low_
1	Left level II lymph node	Out‐of‐field	94.8	81.3	93.9	83.9
2	Inferior left nasopharynx	In‐field	100.0	99.5	100.0	100.0
3	Left lower neck posterior to the sternocleidomastoid muscle	Marginal	100.1	100.0	100.0	100.0
4	Left IIA and IV lymph nodes	In‐field, In‐field	101.8	101.6	99.0	99.0
5	Left I and II lymph nodes	In‐field	100.0	100.0	100.0	99.5
6	Floor of mouth and left neck base	Marginal, Marginal	100.0	101.9	100.0	99.9
7	Floor of mouth and right tongue	In‐field, In‐field	100.0	100.9	100.0	99.9
8	Posterior nasopharyngeal abutting the skull base and clivus	In‐field	97.2	100.6	95.5	98.9
9	Left II and IV lymph nodes	In‐field	98.5	94.8	100.0	99.4
10	Left buccal space	In‐field	100.5	100.9	100.0	97.9

D_95%_, the dose covering 95% of the PTV; PTV, planning target volume; V_95%_, the subvolume of the PTV receiving 95% of the prescription dose.

To facilitate spatial association, the PET/CT scan was registered to the planning CT using landmark‐based rigid registration in Eclipse^TM^.^19^ The registration uncertainty study was not performed and will be the research topic of the future study. However, the image registration was reviewed and approved by the treating physician. The UVN (standard method) and UVC (robustness method) were then examined for spatial association with LF. If there was overlap of at least 0.5 cm^3^ between the underdosed volume and the region of LF, the variable indicating association between the underdosed volumes and LF was set to be TRUE; otherwise it was set to be FALSE (Table 3). The value of 0.5 cm^3^ is chosen to have enough voxels to minimize the possible random errors. The association was further reviewed and approved by an experienced radiation oncologist.

**Table 3:** Association between the underdosed volumes and local failure

Patient	Association between UVN and LF	Association between UVC and LF
1	False	False
2	False	True
3	False	True
4	False, True	False, True
5	True	True
6	False, False	True, True
7	False, False	True, True
8	False	True
9	True	True
10	False	True

UVN, the underdosed volume on the nominal dose distribution; UVC, the underdosed volume on the coldest dose distribution; LF, local failure.

### 2.E Visualization of dose variation in the PTVs

The patient setup uncertainties may not only perturb the dose at the edge of PTVs, but also perturb the dose far from the edge of PTVs. To visualize the dose variation of VMAT plans in the PTVs in the face of patient setup uncertainties, we used the root‐mean‐square dose deviation (RMSD) robustness quantification technique proposed by Liu et al.^20^, ^21^, ^22^ A similar concept of error‐bar volume histogram was proposed by Albertini et al.^23^ The RMSD of voxels was calculated as the square root of the sum square of the differences between the dose calculated under the uncertainty scenarios and the nominal scenario and the mean dose of those seven doses. The calculated RMSD dose file was imported back to Eclipse^TM^ and visualized.

### 2.F Statistical analyses

The null hypothesis was that the underdosed volume was independent of LF. Therefore, the binomial distribution with a probability of 0.5 was used to calculate the p value for the statistical significance of the association between the underdosed volume and LF. We further tested the relationship between the aforementioned association and tumor histology, location, stage, CTV_High_ volume, and treatment modality by using the Fisher's exact test of independence. The 2 × 2 exact contingency table was used.

For the analysis of the Fisher's exact test of independence, the patients were divided into two groups according to tumor location (oral cavity/oropharynx or not), stage (advanced stage or not), CTV_High_ volume (> 100 cc or not), and treatment modality (with surgery or not).

## 3 RESULTS

**Figure 1:**
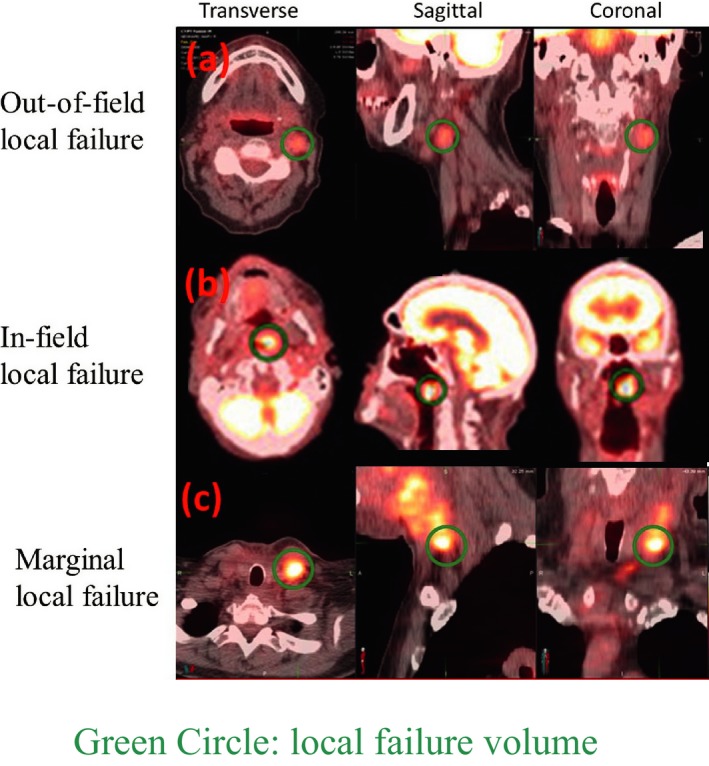
PET/CT scans showing out‐of‐field (*top panel*), in‐field (*middle panel*), and marginal local failure (*bottom panel*) in the transverse (*left column*), sagittal (*middle column*), and coronal (*right column*) plane. The dark green circles indicate the locations of local failure.

Figure 1 shows the PET/CT scans of patients with typical out‐of‐field (*top panel*), in‐field (*middle panel*), and marginal LF (*bottom panel*) in the transverse (*left column*), sagittal (*middle column*), and coronal (*right column*) plane. The dark green circles indicate the locations of LF. The individual LF locations are listed in Table 2. Of these 13 LFs, nine were in‐field, three were marginal, and one was out‐of‐field LFs (Table 2).

The D_95%_ and V_95%_ of PTV_High_ and PTV_Low_ in the nominal dose distribution are listed in Table 2. Although the DVH indices for most patients suggested sufficient PTV coverage (≥ 99%),^24^ all patients had developed LFs. The PTV coverage of patients #1, 8, 9, and 10 in the nominal dose distribution was compromised because of nearby organs at risk.

**Figure 2:**
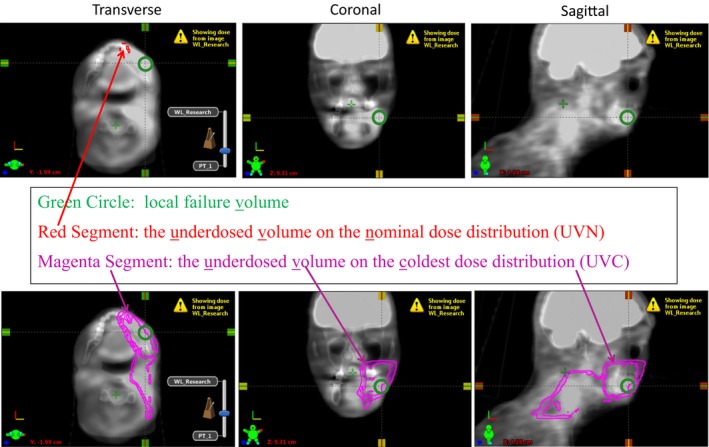
Illustration of the PET/CT fused with the planning CT for one patient: (a) association between the underdosed volume in the nominal dose distribution (UVN) and local failure; (b) association between the underdosed volume in the coldest dose distribution (UVC) and local failure. The local failure was indicated using green circles, while the underdosed volumes were indicated using magenta lines.

Figure 2 shows the PET/CT scan overlaid with the planning CT for patient 6. The LF is indicated with green circles, while the underdosed volumes are indicated with magenta lines. This patient did not have overlap between UVN (standard determination of cold spots) and LF, yet there was overlap between UVC (robustness determination of cold spots) and LF.

The association between the underdosed volumes and LF for all patients is included in Table 3. Of the 13 LFs, three overlapped with UVN, and 11 overlapped with UVC. Of the four patients with compromised PTV coverage, only one patient had overlap between UVN and LF; however, three had the overlap between UVC and LF.

The *p* values from the binominal distribution were calculated to be 0.99 for the association between UVN and LF and .011 for the association between UVC and LF. We also tested the relationship of the association between UVC and LF with tumor location, stage, CTV_High_ volume, and treatment modality. The *P* values from the Fisher's exact test of independence were 0.99, 0.99, 0.99, and 0.44 respectively (Table S2).

**Figure 3:**
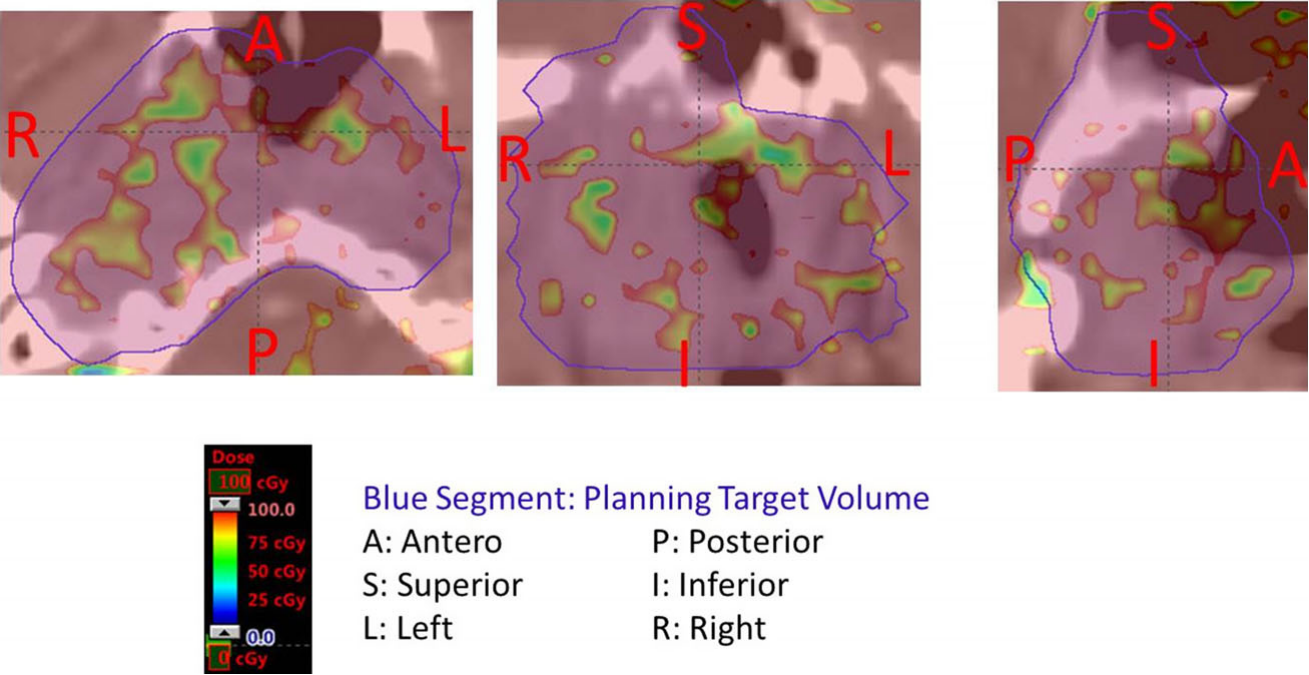
Dose perturbation due to patient setup uncertainties in three planes: (*left*) transversal; (*middle*) frontal; (*right*) sagittal. Patient setup variation appeared to perturb the VMAT dose distribution in the middle of PTVs. The dose perturbation, although small, demonstrated that the static dose cloud approximation is not rigidly satisfied in VMAT.

To show dose variation in the PTVs, the RMSD distributions in three planes calculated from the perturbed doses corresponding to different scenarios of patient‐setup uncertainty are shown in Fig. 3. Patient setup variation appeared to perturb the VMAT dose distribution in the middle of PTVs.

## 4 DISCUSSION

Many factors contribute to LF in radiotherapy including biological factors, volume of disease,^4^ inadequate margins,^4^, ^9^ and inaccurate dose calculation because of inhomogeneity in H&N cancer. In this study, we retrospectively investigated the association of LF identified by PET/CT with two different types of underdosed volumes: (a) UVN, cold spots in PTVs resulting from the compromised PTV coverage on standard VMAT plans and (b) UVC, the underdosed volumes in the coldest dose distribution resulting from patient setup uncertainties on VMAT plans assessing robustness.

One of the major causes for LFs is the suboptimal target delineation.^4^, ^9^ The scope of this work is limited to show which is more relevant to cause the LF due to patient setup uncertainty between the compromised PTV coverage and the potential target underdosage subject to patient setup uncertainties. In this study all the targets were contoured by an experienced radiation oncologist and then reviewed by an independent experienced radiation oncologist. And an experienced radiation oncologist carefully reviewed all the patients with LF and chose patients, who were least likely to have insufficient target delineation. That is also the reason why none of the patients included in this study had the radiation target volumes cropped due to the protection of the nearby organs at risks. Some PTVs were cropped at least 3 mm from the skin if needed due to the imperfection in the dose calculation of our treatment planning system. Fortunately, none of the LFs of the patients included in this study took place in these cropped volumes.

D_95%_ and V_95%_ of PTVs are parameters that are routinely used to evaluate adequacy of target dose coverage. The tumor control probability can be calculated based on various models by using PTV coverage.^25^ Interestingly, six patients (Table 2) had excellent D_95%_ and V_95%_ for both PTV_High_ and PTV_Low_. Of the four patients with relatively less optimal D_95%_ and V_95%_, the LF region spatially overlapped with UVN in one patient; however, it overlapped with UVC in the other three patients. For all 10 patients, the association between LF and UVC was statistically significant (*P* = 0.011), yet the association between LF and UVN was not (*P* = 0.99) (Table 3). This finding suggests that compared to the compromised PTV coverage due to patient setup uncertainty (an important LF indicator), the potential target underdosage subject to patient setup uncertainties is more relevant at least for the H&N cancer patients treated by VMAT included in this study.

Our study showed that most LFs occur within PTV_High_ and PTV_Low_ (PTV_High_ resides in PTV_Low_). Most LFs in our study were in‐field, which is consistent with previous reports (see the references included in Fried et al.,^4^). It appears that patient‐setup uncertainty perturbs the high/low prescription iso‐dose lines and shrinks the volumes enclosed by the corresponding prescription iso‐dose lines. The resulting underdosed volumes formed the region of possible LF within PTV_High_ and PTV_Low_.

Figure 3 showed the dose perturbation due to patient setup uncertainties within PTV for one typical patient. Patient setup variation appeared to perturb the VMAT dose distribution not only at the edge of PTVs but also in the middle of PTVs. Although insignificant, there is measurable dose variation within both PTV_High_ and PTV_Low_. This suggests that the static dose cloud approximation is not strictly valid in VMAT. This first order dose perturbation makes the concept of the compromised PTV coverage different from the concept of the potential target underdosage subject to patient setup uncertainties, although both are trying to account for patient setup uncertainties. This is also the possible reason why the potential target underdosage subject to patient setup uncertainties is more relevant to LFs compared to the compromised PTV coverage.

Therefore, in addition to assessing the D_95%_ and V_95%_ of PTVs, evaluation of the VMAT plan robustness appears to be important for minimizing LFs when treating H&N cancer. The local failures occurred to be significantly associated spatially with the site of underdosage when evaluating the VMAT plan specifically for robustness to patient setup uncertainties. The area of underdosage using the standard method was not significantly associated spatially with the site of local failure.

This study focused on factors inherent in the physical characteristics of VMAT that could potentially contribute to LF. Because of the highly conformal dose distribution of VMAT, a greater potential exists for marginal misses. Therefore, accuracy in target delineation and adequacy of PTV margins are especially important when using VMAT to treat patients with H&N cancer. Small degrees of uncertainty (≤ 3 mm in any direction) appear to lead to LF and should be accounted for with robust planning. Recently, several groups have proposed using robust probabilistic planning to replace the concept of PTV in photon therapy.^26^, ^27^, ^28^, ^29^ The plan robustness quantification tools developed for this study could help design knowledge‐based anisotropic margins in our clinical practice. In addition, the knowledge acquired from this study might aid in further development of stringent image‐guided radiotherapy and immobilization devices, as well as optimal motion management strategies for using VMAT to treat patients with H&N cancer.

Our study had limitations, including a small sample size. We did use the analysis of the Fisher's exact test of independence to check the dependence of the conclusions on tumor location, stage, volume, and treatment modality. The *P*‐values are included in the Table S2. Based on this small patient population, it seems that the conclusion is independent of tumor location, stage, volume, and treatment modality. However, a larger patient population is warranted to verify the clinical relevance of our brute force method, which will be included in our future study. Our patient cohort was also limited in disease subsites. All of the patients had the disease below the eye level. Fried et al.,^4^ reported that in case of sinonasal malignancies, with the disease located at or above the eye level, target coverage was often compromised to protect the critical normal tissue adjacent to the orbit and base of skull, thus increasing the risk of LF in this area. Sinonasal cancer is an uncommon malignancy and we did not have any LFs in the recent past using VMAT that could be included for this study.

In summary, our work alerts radiotherapy practitioners not to rely solely on the standard method for assessment of VMAT PTV coverage to assess target control. The plan robustness analysis appears to be a significantly more important factor associated with LF than the conventional PTV underdosage measures for patients with H&N cancer treated with VMAT. A larger patient population study with a control group would be helpful for further investigation.

## ACKNOWLEDGMENTS

This research was supported by the National Cancer Institute (NCI) Career Developmental Award K25CA168984, the Fraternal Order of Eagles Cancer Research Fund Career Development Award, the Lawrence W. and Marilyn W. Matteson Fund for Cancer Research, Mayo Arizona State University Seed Grant, and the Kemper Marley Foundation.

## CONFLICTS OF INTEREST

None.

## Supporting information

Table S1 Dose volume constrains for H&N radiation treatment from our institution protocol.
Table S2 *P*‐value for the association between the underdosed volume and recurrence volume from the binomial distribution and the relationship between the association with tumor location, tumor stage, CTV_High_ volume, and treatment modality derived from the Fisher's exact test of independence.
