# Supplementary material for: Exploratory study of the association of volumetric modulated arc therapy (VMAT) plan robustness with local failure in head and neck cancer
Source: J Appl Clin Med Phys. 2017 May 14;18(4):76–83. doi: 10.1002/acm2.12099 (PMC5500391; doi:10.1002/acm2.12099)
Supplement: Supplementary file 1 — Table S1 Dose volume constrains for H&N radiation treatment from our institution protocol. Table S2 P‐value for the association between the underdosed volume and recurrence volume from the binomial distribution and the relationship between the association with tumor location, tumor stage, CTVHigh volume, and treatment modality derived from the Fisher's exact test of independence. [file ACM2-18-76-s001.docx]

| **Supplemental Table 1: Dose volume constrains for H&N radiation treatment from our institution protocol.** |
| --- |

| PTV_High_ | [D1% [%]](about:blank) | ≤110% |
| --- | --- | --- |
|  | [D95% [%]](about:blank) | ≥100% |
|  | [V115% [cc]](about:blank) | <0.5 cc |
| CTV_High_ | [V100% [%]](about:blank) | ≥98% |
| Brain | \| [Max [Gy]](about:blank) \| <56 Gy \| \| --- \| --- \| \| [V60 Gy [cc]](about:blank) \| <1 cc \| | |
| Brainstem | \| [Max [Gy]](about:blank) \| ≤50 Gy \| \| --- \| --- \| \| [V30 Gy [%]](about:blank) \| <33% \| | |
| Cord | \| [Max [Gy]](about:blank) \| ≤45 Gy \| \| --- \| --- \| | |
| Cochlea | \| [Mean [Gy]](about:blank) \| <45 Gy \| \| --- \| --- \| | |
| Optic nerve | \| [Max [Gy]](about:blank) \| ≤50 Gy \| \| --- \| --- \| \| [V54 Gy [cc]](about:blank) \| ≤0.1 cc \| | |
| Optic chiasm | \| [Max [Gy]](about:blank) \| ≤50 Gy \| \| --- \| --- \| \| [V54 Gy [cc]](about:blank) \| ≤0.1 cc \| | |
| Eye | \| [Mean [Gy]](about:blank) \| ≤30 Gy \| \| --- \| --- \| \| [V50 Gy [cc]](about:blank) \| ≤0.1 cc \| \| [V40 Gy [%]](about:blank) \| ≤50% \| | |
| Lacrimal | Max [Gy] | ≤30 Gy |
|  | Mean [Gy] | ≤10 Gy |
| Parotid | \| [Mean [Gy]](about:blank) \| <26 Gy \| \| --- \| --- \| \| [V30 Gy [%]](about:blank) \| ≤50% \| \| [V40 Gy [%]](about:blank) \| <33% \| | |
| Oral cavity | \| [Mean [Gy].](about:blank) \| ≤50 Gy \| \| --- \| --- \| \| [V30 Gy [%]](about:blank) \| ≤65% \| \| [V35 Gy [%]](about:blank) \| ≤35% \| | |
| Nasal cavity | \| [Mean [Gy]](about:blank) \| ≤50 Gy \| \| --- \| --- \| \| [V30 Gy [%]](about:blank) \| ≤65% \| \| [V35 Gy [%]](about:blank) \| ≤35% \| | |
| Mandible | \| [Max [Gy]](about:blank) \| ≤70 Gy \| \| --- \| --- \| \| [V75 Gy [cc]](about:blank) \| ≤1 cc \| | |
| Larynx | \| [Max [Gy]](about:blank) \| <60 Gy \| \| --- \| --- \| \| [Mean [Gy]](about:blank) \| ≤35 Gy \| \| [V50 Gy [%]](about:blank) \| ≤27% \| | |
| Esophagus | \| [Mean [Gy]](about:blank) \| ≤34 Gy \| \| --- \| --- \| \| [V35 Gy [%]](about:blank) \| ≤50% \| \| [V55 Gy [%]](about:blank) \| ≤40% \| \| [V70 Gy ≤[%]](about:blank) \| ≤20% \| | |

Abbreviations: CTV, clinical target volume; DVH, dose-volume histogram; PTV, planning target volume.

**Supplemental Table 2: P-value for the association between the underdosed volume and recurrence volume from the binomial distribution and the relationship between the association with tumor location, tumor stage, CTV_High_ volume, and treatment modality derived from the Fisher’s exact test of independence.**

|  | Association between UV and recurrence volume | Tumor location | Tumor stage | CTV_high_ Volume | Treatment modality |
| --- | --- | --- | --- | --- | --- |
| P-value | 0.011 | 0.99 | 0.99 | 0.99 | 0.44 |
